# euL1db: the European database of L1HS retrotransposon insertions in humans

**DOI:** 10.1093/nar/gku1043

**Published:** 2014-10-28

**Authors:** Ashfaq A. Mir, Claude Philippe, Gaël Cristofari

**Affiliations:** 1 INSERM, U1081, Institute for Research on Cancer and Aging of Nice (IRCAN), F-06100 Nice, France; 2 CNRS, UMR 7284, Institute for Research on Cancer and Aging of Nice (IRCAN), F-06100 Nice, France; 3 Faculty of Medicine, Institute for Research on Cancer and Aging of Nice (IRCAN), University of Nice-Sophia-Antipolis, F-06100 Nice, France

## Abstract

Retrotransposons account for almost half of our genome. They are mobile genetics elements—also known as jumping genes—but only the L1HS subfamily of Long Interspersed Nuclear Elements (LINEs) has retained the ability to jump autonomously in modern humans. Their mobilization in germline—but also some somatic tissues—contributes to human genetic diversity and to diseases, such as cancer. Here, we present euL1db, the European database of L1HS retrotransposon insertions in humans (available at http://euL1db.unice.fr). euL1db provides a curated and comprehensive summary of L1HS insertion polymorphisms identified in healthy or pathological human samples and published in peer-reviewed journals. A key feature of euL1db is its sample-wise organization. Hence L1HS insertion polymorphisms are connected to samples, individuals, families and clinical conditions. The current version of euL1db centralizes results obtained in 32 studies. It contains >900 samples, >140 000 sample-wise insertions and almost 9000 distinct merged insertions. euL1db will help understanding the link between L1 retrotransposon insertion polymorphisms and phenotype or disease.

## INTRODUCTION

Repetitive DNA accounts for half of our genome. Most of these repeats are retrotransposons, i.e. mobile genetic elements, which proliferate through an RNA-mediated copy-and-paste mechanism, called retrotransposition. A tiny fraction of human retrotransposons is still able to autonomously generate new copies in modern humans (1). These active elements all belong to the L1HS subfamily (HS stands for human-specific), a subgroup of the L1 (Long Interspersed Nuclear Element-1 or LINE-1) clade of non-Long Terminal Repeat (LTR) retrotransposons found in vertebrates, plants and fungi. The L1 retrotransposon machinery is also able to mobilize *in trans* non-autonomous retrotransposons belonging to the Short Interspersed Nuclear Element (SINE) class (*Alu*, SVA); or cellular RNAs (U6, mRNA), which results in processed pseudogene formation (see (2–4) for recent reviews). Other transposable elements are molecular fossils and do not mobilize in modern humans.

A full-length human L1 is ∼6.0 kb in length, contains an internal promoter located in the 5′-untranslated region and encodes two proteins, ORF1p and ORF2p, both being required for L1 retrotransposition. ORF1p is an RNA-binding protein (5) and ORF2p an enzyme with endonuclease and reverse transcriptase activities (6,7). These proteins associate with the L1 mRNA to form a ribonucleoprotein particle, which is considered as the core of the L1 retrotransposition machinery (8,9). A new L1 copy is produced when ORF2p nicks the genomic DNA and extends this newly formed 3′ end using the L1 mRNA as a template, a process known as target-primed reverse transcription (TPRT) (7,10,11). This process results in a short duplication of the target site (TSD, target-site duplication). Abortive retrotransposition often leads to 5′ truncated L1 copies (12,13). Some L1 insertions exhibit both a 5′ truncation and an inversion, due to twin priming (14). Finally, L1 insertions can also contain 5′- or 3′-transductions corresponding to genomic sequences immediately upstream or downstream their progenitor copies. Such events originate from the retrotransposition of L1 transcripts generated from upstream promoters or ending downstream of the L1 sequence due to the weakness of the natural L1 polyadenylation signal (13,15,16). L1 target site preference is currently not fully defined, but both the endonuclease consensus sequence and the ability of the target site to partially anneal to the L1 mRNA poly(A) tail contribute to this process (7,17–19).

In the past 5 years, advances in deep-sequencing technologies have shed a new light on the extent of L1-mediated genome variation (20,21). L1HS represents ∼3.3 Mb of the reference human genome (∼0.1%). These L1 copies are often referred to as ‘reference L1HS elements’. However, each individual has additional L1HS copies not present in the reference genome, referred to as ‘non-reference L1HS elements’, which contribute to our genetic diversity (22–27). On the average, two human individual genomes differ at 285 sites with respect to L1 insertion presence or absence (27). These recent studies have also led to the discovery that L1HS is not only able to mobilize in the germline—resulting in inheritable genetic variations (3,28,29)—–but can also jump in some somatic tissues, such as brain (30–32) or in many cancers (26,33–39). Most retrotransposition events are the consequence of highly active, or ‘hot’, L1HS loci that constitute a small minority of full-length L1HS elements, with many of these being population-specific or even unique to a particular individual (private copies) (1,24). Therefore, understanding the link between L1HS insertion polymorphisms and phenotype or disease requires a comprehensive view of the different L1HS copies present in given individuals.

euL1db provides a curated and comprehensive summary of L1 retrotransposon insertion polymorphisms (RIPs) identified in healthy or pathological human samples and published in peer-reviewed journals. A sample is defined here as the primary biological material (e.g. tissue biopsy, blood, cell or cell line) from which a genomic DNA preparation was obtained and a sequencing library prepared. An important feature of euL1db is that insertions can be retrieved at a sample-by-sample level to facilitate correlations between the presence/absence of an L1 insertion with a specific phenotype or disease.

## DATABASE STRUCTURE AND CONTENT

**Figure 1.**
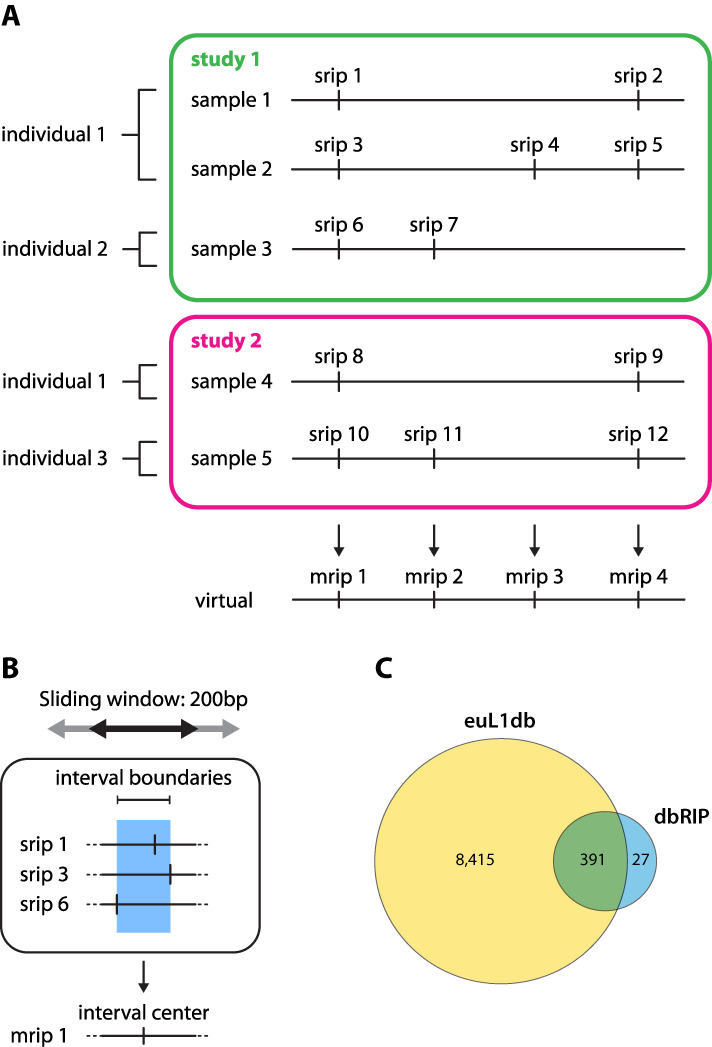
Database organization, data model and content. (**A**) Relationship between euL1db objects. euL1db is organized by study. Each study contains one or more samples. A sample originates from a single individual. Individuals can be analyzed in multiple studies. An SRIP (sample retrotransposon insertion polymorphism) is a real insertion detected in a given sample and has a unique ID prefixed by srip. Several samples from different individuals might possess an SRIP at the same genomic location. A private L1HS insertion will correspond to an SRIP only found in samples of the same individual. Inversely, an L1HS insertion which is fixed in the human population will appear as an SRIP at the same location in all the genome-wide samples of euL1db. Thus SRIP are highly redundant. In contrast, MRIP (meta-retrotransposon insertion polymorphisms) are virtual insertions obtained by merging overlapping or close SRIP, which are likely to correspond to the same retrotransposition event. Thus MRIP are non-redundant. (**B**) Approach used in euL1db to define unique L1HS insertion events. Nearby SRIPs are merged into a single MRIP if they satisfy all the following requirements: (i) they are located within 200 bp of each other, (ii) they share the same strand orientation, and (iii) they are all germline. Somatic retrotransposition events are unique by nature, and are not merged with germline events, nor merged together. Therefore, somatic SRIPs give rise to MRIPs containing only a single SRIP. (**C**) Overlap between euL1db and dbRIP. Numbers correspond to MRIP records in euL1db and to L1HS records in dbRIP (transposable elements not belonging to the L1HS subfamily were not taken into account to draw this Venn diagram).

The euL1db database is organized in several tables, which are interconnected in a dynamic way, through the MySQL relational database management system. A simplified view of the object relationships is depicted in Figure 1 and a more detailed view of the underlying database structure is shown in Supplementary Figure S1.

The ‘Study’ table contains information about the study in which L1HS insertions were cataloged and mapped. Typically, a study will correspond to a single publication. Each study uses a coherent set of methods and analyses. Because these parameters determine to a large extent the variability that exists between data sets, all data in euL1db are organized by study. The ‘Individual’ table relates to the source individuals from whom the samples were originally taken from. The same individual might have been subjected to multiple analyses, possibly in different studies (e.g. Figure 1, individual 1, present in study 1 and 2). When available, euL1db stores the gender, the geographical origin, potential familial links with other individuals in euL1db and health information. All individuals from the 1000 Genomes Project have been incorporated in euL1db, even though only a small portion has been analyzed for L1HS content. This was necessary to maintain the family architecture and to facilitate future updates. The ‘Sample’ table describes the primary biological sample taken from a given individual and from which L1HS insertions were cataloged and mapped. When available, euL1db stores the anatomical and potential pathological data, and whether it was prepared from a single-cell or from multiple cells. Potential relationships between samples are also recorded (e.g. normal-tumor pairs). Importantly, a given sample can only be linked to a single study, and is given a unique ID. Unanalyzed individuals from the 1000 Genomes Project are not linked to any sample.

L1HS insertions found in a given sample are cataloged as ‘sample retrotransposon insertion polymorphism’ or SRIP. An SRIP is defined minimally by its genomic coordinates and is linked to a unique sample (Figure 1A). Additional optional information might include its genomic strand, its internal sequence, the length and sequence of its TSD or deletion, the presence of a 5′- or 3′-transduction, the presence of a 5′ inversion, the size of its downstream poly(A) sequence, its coordinates relative to the Repbase L1HS consensus sequence (and the positions of an inversion, if present), its allele frequency, if it is a somatic or a germline insertion, and its integrity (i.e. full length, 5′-truncated, 3′-truncated or internal fragment). Each SRIP is given a unique ID in euL1db, which is prefixed by srip (e.g. srip34564). Because several SRIP might actually correspond to the same original insertion event, some have identical or close genomic coordinates (e.g. srip 1, 3, 6, 8 and 10 in Figure 1A). To reduce this redundancy and to facilitate comparisons within and across studies, a set of virtual insertions named ‘meta-retrotransposon insertion polymorphism’ or MRIP has been computationally generated (Figure 1A and B). An MRIP refers to a unique genomic interval, which contains overlapping or close SRIP, likely corresponding to the same original insertion event. In practice, nearby SRIPs are merged into a single MRIP if they satisfy all the following requirements: (i) they are located within 200 bp of each other, (ii) they share the same strand orientation, and (iii) they are all germline insertions. Somatic retrotransposition events are unique by nature, and are not merged with germline events, nor merged together. Therefore, somatic SRIPs give rise to MRIPs containing only a single SRIP. Using a 200-bp window around SRIP rather than precise coordinates was necessary since different methods and studies have variable accuracy in defining the precise location of L1HS insertions. The rational for choosing the size of this window is detailed in the Supplementary Methods. Each MRIP is given a unique ID in euL1db, which is prefixed by mrip (e.g. mrip1234). Although the probability of finding two independent germline insertion events in the same 200-bp window is extremely low, it is not null. The euL1db framework allows users to compare annotations provided for each SRIP within a given MRIP. Depending on the study, SRIP annotations may include the length and/or sequence of the TSD, the reverse-transcribed L1 sequence or other additional potential rearrangements (inversion, transduction). In a situation where distinct insertion events were wrongly combined in a single MRIP, discrepancies in the SRIPs annotations could alert the user that caution should be taken. This also applies for the most extreme case, i.e. two independent insertion events occurring at the same exact nucleotide. Since reference L1HS insertions are virtual insertions derived from a consensus reference sequence and not from a biological sample, we have chosen to include them in a distinct table, entitled the ‘Reference’ table, and to assign them an ID prefixed by ref (e.g. ref123). This table is used internally to determine whether a given SRIP or MRIP actually corresponds to a reference L1HS insertion, and to annotate each record. The total number of SRIPs and MRIPs included for each study is graphed in Supplementary Figure S3.

In addition to these main tables, euL1db uses a ‘Method’ table, which contains the methods used to call SRIPs in the different studies, and a ‘Family’ table, which classifies the familial relationships between euL1db individuals (mostly from the 1000 Genomes Project). An individual without known relative in euL1db is not linked to any family.

The data contained in euL1db originate from peer-reviewed publications and have been manually curated and entered. The source of data and the curation process are detailed in Supplementary Methods and in Supplementary Table S1. The reference L1HS insertions have been processed from the UCSC RepeatMasker track table. The summary statistics at the time of writing are displayed in Table 1.

**Table 1.** euL1db content statistics

Record type	Number of records
Studies	32^a^
Samples	943
Individuals	741
Families	50^b^
SRIP	142,495
MRIP	8991
Reference L1HS	1545

^a^Out of 32 studies, 10 used high-, 1 medium- and 21 low-throughput approaches.

^b^With at least two individuals analyzed.

## DATA ACCESS

euL1db can be interrogated through a user-friendly Web Server (http://euL1db.unice.fr). A set of detailed tutorials and examples of use are accessible from the ‘Help’ tab. The detailed description of the Web Server architecture is described in Supplementary Figure S1.

There are several ways to query euL1db: (i) by searching SRIP or MRIP located in a single locus (genomic region, gene) or in a single individual (‘Search’ tab); (ii) by browsing the different tables and using filters to select a specific subset of data across and within studies, families, individuals, samples, insertions (‘Browse’ tab); (iii) by batch query using a list of multiple loci (genomic coordinates) or genes (gene names) (‘Utilities’ tab).

Users can choose to display L1 insertions as SRIP or MRIP in (i) graphical- (UCSC genome browser, dbVar genome browser) (40,41); (ii) tabular- (sortable html tables); or (iii) text-formats (including in standard BED format for subsequent analyses with other tools). Tables can be customized to display the information of interest for the user.

## RELATIONSHIP AND DIFFERENCES WITH OTHER DATABASES

Several resources are related to—but distinct from—euL1db. Repbase is a database of consensus repetitive DNA sequence and as such does not contain any localization information (42). One of its entries is the L1HS consensus sequence and has been subsequently used to annotate the human reference genome and to identify the genomic loci corresponding to L1HS elements (Smit, A.F.A., Hubley, R. & Green, P. *RepeatMasker Open-3.0*. 1996–2010 <http://www.repeatmasker.org>). This information is available through the RepeatMasker table of the UCSC Genome Browser (40). The reference L1HS elements included in the ‘Reference’ table of euL1db have been processed and annotated using the latter. dbRIP was an early effort to catalog and annotate polymorphic retrotransposon insertions in humans (43). In contrast to dbRIP, euL1db stores data in a sample-wise manner and contains the most recent data sets obtained by high-throughput sequencing, including those from the 1000 Genomes Project. Although dbRIP could not be directly included in euL1db since samples are not documented in dbRIP, 94% of dbRIP L1HS records have an MRIP equivalent in euL1db (Figure 1C). dbRIP has also unique features since it contains non-autonomous human retrotransposons such as Alu or SVA sequences and not only L1HS insertions. As a particular case of structural variation, L1 retrotransposon insertions are also documented in dbVar/DGVa or DGV as mobile element insertions (MEI) (41). The data structure logics in dbVar/DGVa and euL1db are comparable (sample-wise variants and merged variants). euL1db is specialized for L1HS insertions, while dbVar/DGVa can include any type of structural variants, including MEI. However, the set of information for L1HS insertions provided by euL1db is much more exhaustive, and only a single study (out of 32 at the time of writing) stored in euL1db was also deposited in dbVar/DGVa.

## CONCLUDING REMARKS

High-throughput sequencing technologies have considerably fostered the study of L1-mediated genomic variation and its impact on human health. We anticipate that this trend will continue in the next years, particularly with the availability of long-reads sequencing approaches, which might greatly facilitate the detection of L1HS insertions and their accurate positioning on the genome by generating reads that span the entire element and both flanking regions. In this respect, euL1db database and server have been tailored to support a considerable increase of SRIP, while keeping a fast-response time. To summarize, euL1db provides a centralized and user-friendly access to known germline and somatic L1HS insertions, which will be critical to elucidate the physiological or pathological impact of novel L1HS insertions. This resource will be useful in a large variety of fields such as human genetics, neurosciences or cancer genomics.

## SUPPLEMENTARY DATA

Supplementary Data are available at NAR Online.
